# Mesenchymal Stem Cells Inhibit the Effects of Dexamethasone in Multiple Myeloma Cells

**DOI:** 10.1155/2022/4855517

**Published:** 2022-04-04

**Authors:** Mingyang Deng, Huan Yuan, Hongling Peng, Sufang Liu, Xiang Xiao, Zhihua Wang, Guangsen Zhang, Han Xiao

**Affiliations:** ^1^Department of Hematology, The Second Xiangya Hospital, Central South University, Changsha 410011, China; ^2^Department of Neurology, The Second Xiangya Hospital, Central South University, Changsha 410011, China

## Abstract

Mesenchymal stem cells (MSCs) participate in the occurrence and development of multiple myeloma. This study is aimed at exploring whether the presence of MSCs affects dexamethasone's antitumor effects against multiple myeloma. Multiple myeloma cells (OPM-2 and RPMI8226 cells) were cocultured with MSCs with or without dexamethasone. Cell viability was determined by using cell number count, 3-(4,5-dimethylthiazol-2-yl)-2,5-diphenyltetrazolium bromide assay, and colony formation assay, respectively. Cell cycle distribution and cell apoptosis were evaluated by using flow cytometry. The mRNA and protein expressions of target genes were checked by using qRT-PCR and western blotting, respectively. It was found that cell viability of multiple myeloma cells increased in the presence of MSCs. Besides, the presence of MSCs suppressed cell apoptosis induced by dexamethasone via the regulation of BCL-2 (B cell lymphoma 2). The presence of MSCs also affected the effects of dexamethasone on cell cycle distribution. Similarly, LINC00461 overexpression suppressed the inhibition of cell proliferation, suppressed the induction of cell apoptosis, and affected the effects on cell cycle distribution induced by dexamethasone insult. However, LINC00461 knockdown enhanced the inhibitory effects on cell proliferation and the induction of cell apoptosis induced by dexamethasone. In summary, MSCs inhibited the effects of dexamethasone on multiple myeloma and its regulatory effects were associated with LINC00461.

## 1. Introduction

Multiple myeloma is one type of blood cancer which begins in the plasma cells [1, 2]. Multiple myeloma is a fatal plasma disease and remains incurable for most patients, accounting for 10% of hematological malignancies [2]. The occurrence of multiple myeloma weakens the bone tissue, causes bone fractures, and impairs the immune system [2]. The incidence and mortality of multiple myeloma are 160,000 and 106,000 cases, respectively, in 2018 [3]. The International Staging System classified multiple myeloma as three stages, and the median survival rate is lower than 65 months for all stages [4]. Risk factors for multiple myeloma include genetic variants, environmental factors, family history, gender, and age [5]. However, the underlying mechanisms of multiple myeloma are not well elucidated.

A series of therapeutic strategies for multiple myeloma has been employed which includes chemotherapy, radiation therapy, immunotherapy, targeted therapy, bone-modifying therapy, and bone marrow transplantation [6, 7]. Dexamethasone is a corticosteroid and works on the immune system by relieving inflammation [8]. Dexamethasone is also known as one of the most frequently used medications in the treatment of multiple myeloma by preventing the recruitment of blood cells into the lesions where myeloma cells cause damage [9]. The International Myeloma Foundation reported that dexamethasone is regarded as the most effective medication for the treatment of multiple myeloma [10]. In addition to being used alone, the combination of dexamethasone with other medications is also widely used in multiple myeloma [11, 12]. These studies demonstrated that dexamethasone was widely used in multiple myeloma in the 1990s and less commonly used in these years after new agents with different mechanisms of action have been approved for multiple myeloma therapy.

Mesenchymal stem cells (MSCs) are one of the predominant cell populations in the bone marrow stroma [13, 14]. Numerous studies reported that MSCs are closely linked with the occurrence and development of multiple myeloma [15–17]. In the bone marrow environment, MSCs can differentiate into various cell types including osteocytes, osteoblasts, chondrocytes, adipocytes, and fibroblasts [17, 18]. Interestingly, when multiple myeloma occurs, MSCs exhibited different functional modes as normal MSCs [19]. The recruitment of MSCs into the tumor sites promotes tumor growth and metastasis in part by regulating the secretion of exosomes and the release of cytokines [19]. In this regard, it would be of importance to explore the effects of MSCs on dexamethasone-mediated multiple myeloma therapy.

Long intergenic noncoding (Linc) RNAs are noncoding transcripts of more than 200 nucleotides, which have been widely reported to play crucial roles in tumorigenesis, tumor progression, and tumor metastasis [20]. LincRNAs have also been known to be associated with the pathogenesis of multiple myeloma [21]. In 2019, we identified that LINC00461 was highly expressed in multiple myeloma, which was able to promote cell proliferation [22]. Interestingly, our study revealed that LINC00461 is enriched in MSC-derived exosome and knockdown of LINC00461 is effective for suppressing multiple myeloma [22]. Considering the roles of LINC00461 in the MSCs, the present study also explored the effects of LINC00461 overexpression on dexamethasone-mediated multiple myeloma therapy.

## 2. Methods and Materials

### 2.1. Cell Culture

Human multiple myeloma cell lines, OPM-2 and RPMI8226 cells, were obtained from ATCC (Manassas, VA). The cells were maintained in RPMI-1640 medium supplemented with fetal bovine serum (10%, FBS, Gibco, Grand Island, NY) with 5% CO_2_ condition at 37°C. Human MSCs (passages 4-6) were obtained and cultured as previously described [23]. The coculture of MSCs and multiple myeloma cell lines (OPM-2 or RPMI8226) was performed on a 6-well transwell coculture system. Multiple myeloma cell lines were cultured in the inserts, and the MSCs (1 × 10^6^) were cultured in the plate as a nontreatment group. Besides, a final concentration of 10 *μ*M dexamethasone (Sigma, St. Louis, MO) was added to the inserts as the treatment group.

### 2.2. Construction of LINC00461 Overexpression and Knockdown Stable Cell Lines

The cells were cultured in RPMI-1640 medium with 10% FBS supplement with 5% CO_2_ condition at 37°C. When the cells reached a confluency of 60-70%, the cells were then transfected with the pcDNA3.1 plasmids containing the sequence of LINC00461 by using plasmid transfection reagent (Lipofectamine 2000, Thermo Fisher, Waltham, MA). Besides, the cells were transfected with pcDNA3.1 plasmids as the negative control group. Next, the cells were treated with a medium containing 10 *μ*M dexamethasone. In addition, to establish the LINC00461 knockdown cell lines, when the cells reached a confluency of 60-70%, the cells were transfected with LINC00461 siRNA by using transfection reagent. Scrambled siRNA was used as the negative control group.

### 2.3. Determination of Cell Viability

Cell viability was determined by using 3-(4,5-dimethylthiazol-2-yl)-2,5-diphenyltetrazolium bromide (MTT, Sigma), cell number count, and colony formation assays, respectively. After treatment with dexamethasone, MTT reagent (10 *μ*L) was added to each well, and the cells were incubated for 4 h. Detergent reagent was then added to dissolve the purple precipitate, and the cells were incubated in dark at room temperature for another 2 h. The plate was read at a wavelength of 570 nm. Cell number count assay was counted every 24 h by using the cell counter (Beckman, Brea, CA). For cell colony formation assay, after the cells were treated with dexamethasone for 2 weeks, they were stained with 0.5% crystal violet solution. The colonies were further counted using a stereomicroscope.

### 2.4. Measurement of Cell Apoptosis

Annexin V-PI staining was used to determine cell apoptosis as previously reported [24]. After being treated with 10 *μ*M dexamethasone, the harvested cells were stained with Annexin V staining solution (Invitrogen) for 30 min. Next, the cells were centrifuged and the staining solution was removed followed by staining with propidium iodide (PI) staining solution in dark for another 15 min. The cell suspension was examined by using flow cytometry to calculate the percentage of apoptotic cells.

### 2.5. Determination of Cell Cycle Distribution

PI staining was employed to determine cell cycle distribution. After being treated with 10 *μ*M dexamethasone, the fixed cells were washed with phosphate-buffered saline followed by incubating with RNase solution. After fixation, the cells were then stained with PI staining solution in the dark. DNA content was evaluated by using flow cytometry, and the cell cycle distribution was analyzed by using FlowJo.

### 2.6. qRT-PCR

The LINC00461 primers were provided by GenScript (Nanjing, China). Total RNAs were obtained by using TRIzol Reagent (Invitrogen) according to the manufacturers' document. RNAs were precipitated using isopropyl alcohol. After complete removal of the supernatant, RNA washing buffer was then added and vortexed, followed with RNase-free water to elute RNA. RNA concentration and quality were checked by using the NanoDrop instrument. Prior to application of the advanced master mix for quantitative analysis, reverse transcription was conducted to synthesize the cDNA library. The accuracy of the PCR reactions was analyzed by melt curves. The expression level of target gene was calculated by 2-^△△^Ct values.

### 2.7. Western Blotting Assay

Protein extraction was conducted according to published protocol [25]. In brief, the cells were scratched by using a radioimmunoprecipitation assay buffer with a protease inhibitor on ice. Further, the removal of the insoluble components was performed by using centrifugation. Protein concentration was qualified by using a bicinchoninic acid protein assay kit. An equal amount of protein sample (20 *μ*g) was loaded on the precast sodium dodecyl sulfate–polyacrylamide gel electrophoresis gel and then transferred to 0.22 *μ*m polyvinylidene fluoride membranes. The membranes were blocked with 5% bovine serum albumin at room temperature for 2 h. The primary antibodies against *β*-actin (1 : 3000, Sigma) and BCL-2 (1 : 1000, Abcam) were added to incubate with the membranes at 4°C overnight. Next, the membranes were then incubated with appropriate horseradish peroxidase-conjugated secondary antibody at room temperature for another 2 h. The expressions of target proteins were normalized to the *β*-actin.

### 2.8. Statistics

Data, presented as the mean ± SD, was analyzed by using GraphPad Prism 8 (San Diego, CA). One-way or two-way ANOVA was performed to analyze the differences, and a corresponding post hoc test was further performed for intergroup analysis. A *p* value less than 0.05 was considered as a statistical difference.

## 3. Results

### 3.1. MSCs Suppressed the Inhibitory Effects of Dexamethasone on Cell Proliferation

**Figure 1:**
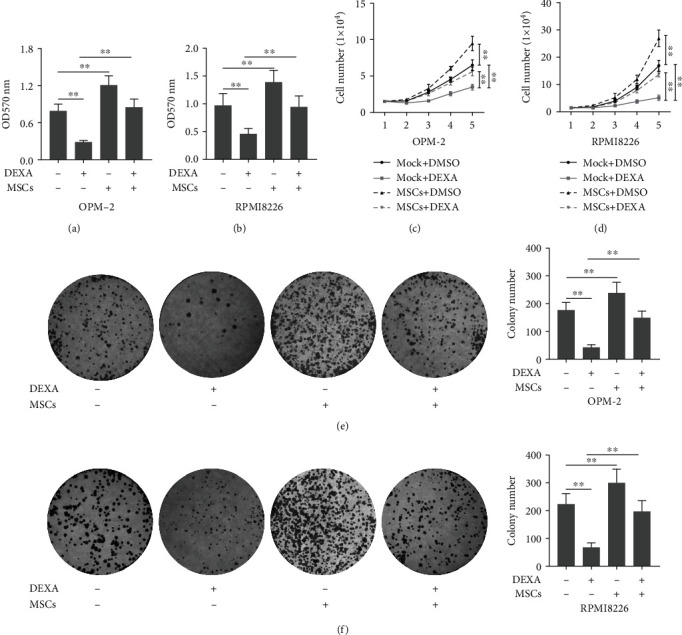
The presence of MSCs suppressed the inhibitory effects of dexamethasone on cell proliferation. (a, b) The OPM-2 or RPMI8226 cells were cocultured with MSCs in a 6-well transwell microplate with or without dexamethasone (10 *μ*M). After 48 hours, cell viability was determined using the MTT assay. Besides, (c–f) cell number and colony formation assays were also determined on the OPM-2 and RPMI8226 cells. Mock indicates that the cells were transfected with a vector. One-way ANOVA for the data in (a), (b), (e), and (f). Two-way ANOVA for the data in (c) and (d). ^∗∗^*p* < 0.01 and ^∗∗∗^*p* < 0.001.

We investigated whether the presence of MSCs affected the effects of dexamethasone on cell proliferation. The results demonstrated that treatment with dexamethasone (10 *μ*M) significantly decreased the cell viability of OPM-2 and RPMI8226 cells (Figures 1(a) and 1(b)). However, the presence of MSCs increased the cell viability of OPM-2 and RPMI8226 cells as compared to the dexamethasone-treated cells (Figures 1(a) and 1(b)).

To confirm this observation, we also used cell number and colony formation assays. It was shown that treatment with dexamethasone (10 *μ*M) significantly decreased the cell number and colony number, respectively (Figures 1(c)–1(f)). Interestingly, the presence of MSCs increased the cell and colony numbers of OPM-2 and RPMI8226 cells as compared to the dexamethasone-treated cells (Figures 1(c)–1(f)).

### 3.2. MSCs Regulated the Effects of Dexamethasone on Cell Apoptosis and Cell Cycle Distribution

**Figure 2:**
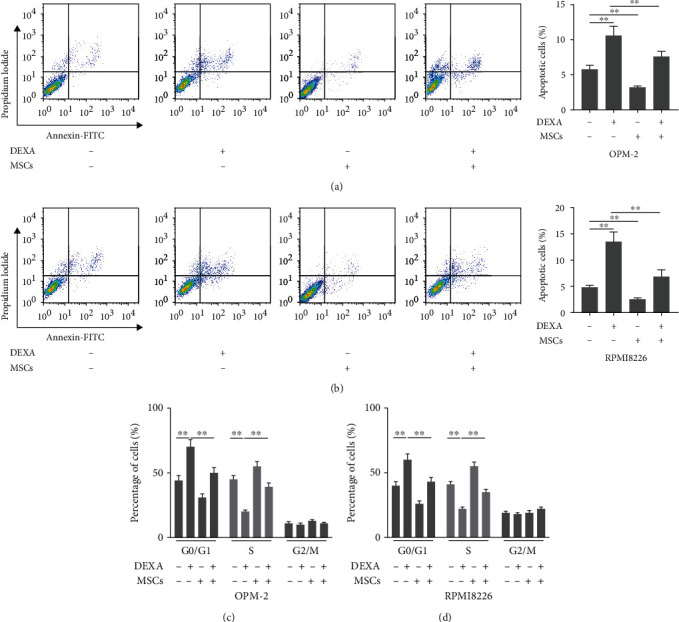
The presence of MSCs regulated the effects of dexamethasone on cell apoptosis and cell cycle distribution. (a, b) The OPM-2 or RPMI8226 cells were cocultured with MSCs in a 6-well transwell microplate with or without dexamethasone (10 *μ*M). After 48 hours, cell apoptosis assay was determined by using flow cytometry. Besides, (c, d) cell cycle distribution was also determined by using flow cytometry assay. One-way ANOVA. ^∗∗^*p* < 0.01 and ^∗∗∗^*p* < 0.001.

In addition to measuring cell viability, we also investigated whether the presence of MSCs affected the effects of dexamethasone on cell apoptosis and cell cycle distribution. The results demonstrated that treatment with dexamethasone (10 *μ*M) significantly increased the percentage of apoptotic OPM-2 and RPMI8226 cells (Figures 2(a) and 2(b)). However, the presence of MSCs decreased the percentage of apoptotic OPM-2 and RPMI8226 cells as compared to the dexamethasone-treated cells (Figures 2(a) and 2(b)). Moreover, we also observed that cells were arrested at the G0/G1 phase in the dexamethasone (10 *μ*M)-treated group (Figures 2(c) and 2(d)). Interestingly, the presence of MSCs increased the percentage of cells in the S phase, while decreased the percentage of cells in the G0/G1 phase.

### 3.3. The Overexpression of LINC00461 Promoted the Protein Expressions of BCL-2

**Figure 3:**
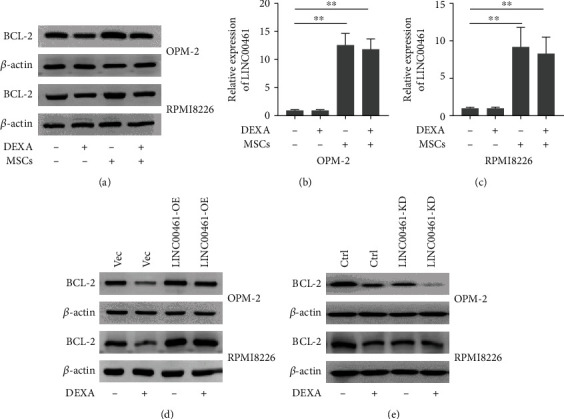
The overexpression of LINC00461 promoted the protein expressions of BCL-2. (a) The OPM-2 or RPMI8226 cells were cocultured with or without MSCs in a 6-well transwell microplate with or without dexamethasone (10 *μ*M). After 48 hours, the protein expressions of BCL-2 were determined in the OPM-2 or RPMI8226 cells by using qRT-PCR and western blotting analysis, respectively. In addition, (b, c) the relative expression of LINC00461 in OPM-2 and RPMI8226 cells was determined by using qRT-PCR. (d) The protein expressions of BCL-2 were determined by using western blot analysis in the normal OPM-2 and RPMI8226 cells and LINC00461 overexpressing cell lines (LINC00461-OE). *β*-Actin was used as an internal control. (e) The protein expressions of BCL-2 were determined by western blot analysis in the normal OPM-2 and RPMI8226 cells and LINC00461 knockdown cells (LINC00461-KD). *β*-Actin was used as an internal control. One-way ANOVA. ^∗∗^*p* < 0.01.

We further investigated the underlying mechanisms of MSCs on the regulation of cell apoptosis by determining the expressions of BCL-2, as the BCL-2 family is the best characterized protein family involved in the regulation of apoptotic cell death. The results showed that treatment with dexamethasone (10 *μ*M) inhibited the expressions of BCL-2, whereas the presence of MSCs increased the expressions of BCL-2 (Figure 3(a)), indicating the presence regulated dexamethasone-induced cell apoptosis in part by the regulation of BCL-2.

In addition, we also examined the mRNA levels of LINC00461. The presence of MSCs significantly increased the mRNA levels of LINC00461 in OPM-2 and RPMI8226 cells (Figures 3(b) and 3(c)). Next, to investigate the roles of LINC00461 in multiple myeloma cells, we successfully constructed the LINC00461 overexpression cell lines, which is supported by the significant increase of mRNA expression levels of LINC00461 in OPM-2 and RPMI8226 cells (Figure 3(d)). When LINC00461 was knocked down, the expression of BCL-2 decreased, and when LINC00461 was knocked down and dexamethasone (10 *μ*M) was added, the expression of BCL-2 decreased more significantly (Figure 3(e)).

### 3.4. The Overexpression of LINC00461 Suppressed the Inhibitory Effects of Dexamethasone on Cell Proliferation

**Figure 4:**
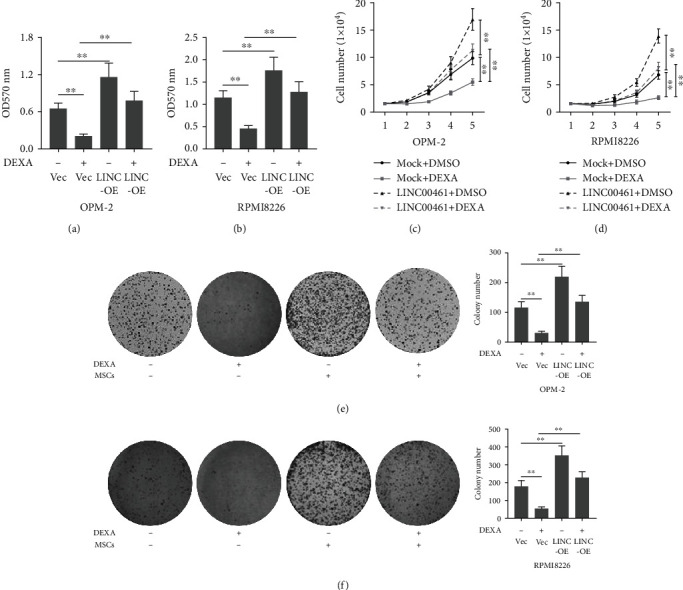
The overexpression of LINC00461 suppressed the inhibitory effects of dexamethasone on cell proliferation. (a, b) Cells were treated with or without dexamethasone (10 *μ*M). After 48 hours, cell viability was determined by using the MTT assay. (c–f) In addition, cell number and colony formation assays were determined on OPM-2 and RPMI8226 cells. Mock indicates that the cells were transfected with a vector. One-way ANOVA for the data in (a), (b), (e), and (f). Two-way ANOVA for the data in (c) and (d). ^∗∗^*p* < 0.01.

Moreover, we investigated whether LINC00461 overexpression affected the effects of dexamethasone on cell viability. LINC00461 overexpression significantly increased the cell viability of OPM-2 and RPMI8226 cells, respectively, as compared to cells transfected with vector (Figures 4(a) and 4(b)). In addition, to confirm this observation, we also determined cell viability by using cell number count and colony formation assay, respectively. Interestingly, the results demonstrated that LINC00461 overexpression significantly increased cell number (Figures 4(c) and 4(d)) and the formation of the colony (Figures 4(e) and 4(f)), respectively.

### 3.5. The Overexpression of LINC00461 Regulated the Effects of Dexamethasone on Cell Apoptosis and Cell Cycle Distribution

**Figure 5:**
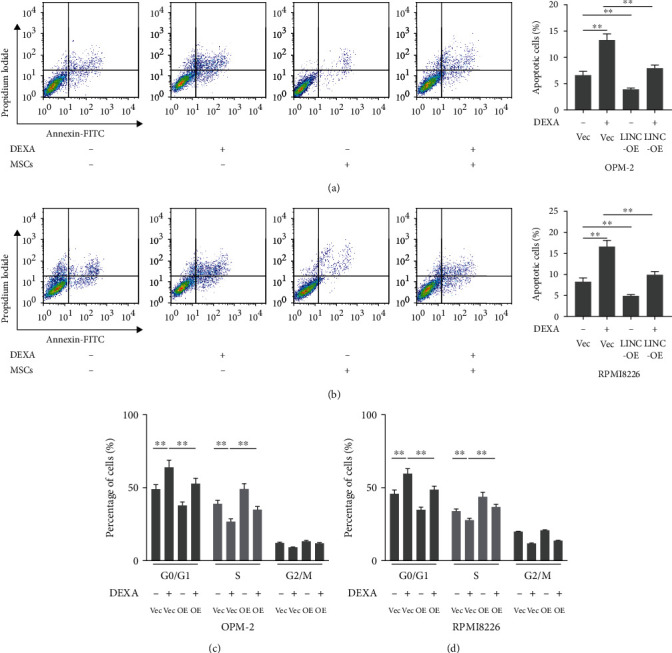
The overexpression of LINC00461 regulated the effects of dexamethasone on cell apoptosis and cell cycle distribution. (a, b) Cells were treated with or without dexamethasone (10 *μ*M). After 48 hours, cell apoptosis assay was determined by using flow cytometry. Besides, (c, d) cell cycle distribution was also determined by flow cytometry. One-way ANOVA. ^∗∗^*p* < 0.01.

We determined whether LINC00461 overexpression affected the effects of dexamethasone on cell cycle distribution and cell apoptosis. As expected, dexamethasone (10 *μ*M) treatment significantly increased the percentage of apoptotic cells and G0/G1 cells. Interestingly, our results showed that LINC00461 overexpression decreased the percentage of apoptotic cells as compared to the dexamethasone-treated cells (Figures 5(a) and 5(b)). In addition, LINC00461 overexpression decreased the percentage of cells in the G0/G1 phase and increased the percentage of cells in the S phase (Figures 5(c) and 5(d)).

### 3.6. The Knockdown of LINC00461 Enhanced the Effects of Dexamethasone on Cell Viability and Apoptosis

**Figure 6:**
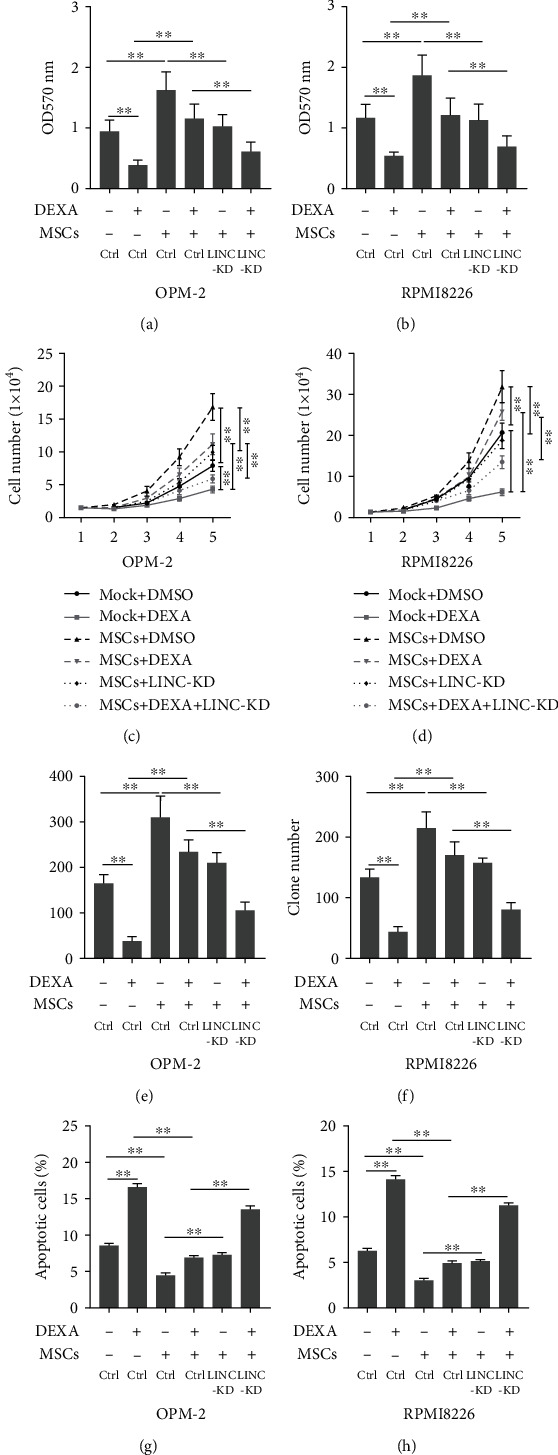
Knocking down of LINC00461 inhibited cell proliferation and promoted apoptosis. (a, b) The OPM-2 or RPMI8226 cells were cocultured with MSCs in a transwell 6-well microplate with or without dexamethasone (10 *μ*M) and transfected the cells with LINC00461 siRNA. After 48 hours, cell viability was determined using the MTT assay. Besides, (c–f) cell number and colony formation assays were also determined on the OPM-2 and RPMI8226 cells. (g, h) The OPM-2 or RPMI8226 cells were cocultured with MSCs in a 6-well microplate with or without dexamethasone (10 *μ*M) and transfected the cells with LINC00461 siRNA. After 48 hours, cell apoptosis assay was determined by using flow cytometry. Ctrl: scramble siRNA. One-way ANOVA for the data in (a), (b), (e), (f), (g), and (h). Two-way ANOVA for the data in (c) and (d). ^∗∗^*p* < 0.01.

Finally, we determined whether the knockdown of LINC00461 affected the effects of dexamethasone on cell apoptosis and viability. Dexamethasone (10 *μ*M) treatment decreased the cell viability of OPM-2 and RPMI8226 cells, respectively. Interestingly, the knockdown of LINC00461 further decreased the cell viability (Figures 6(a) and 6(b)). Consistently, the knockdown of LINC00461 also enhanced the effects of dexamethasone on cell viability by decreasing cell number (Figures 6(c) and 6(d)) and the formation of the colony (Figures 6(e) and 6(f)), respectively. Furthermore, the knockdown of LINC00461 increased the percentage of apoptotic cells as compared to the dexamethasone-treated cells (Figures 6(g) and 6(h)).

## 4. Discussion

In this study, we investigated the roles of MSCs in dexamethasone-mediated antitumor effects against multiple myeloma cells. Our results demonstrated that the presence of MSCs suppressed dexamethasone-mediated antitumor effects, in part, by (1) suppressing its cytotoxic effects, (2) inhibiting its proapoptotic effects, and (3) affecting its regulatory effects on cell cycle distribution. Interestingly, we also revealed that the overexpression of LINC00461 in multiple myeloma cells exhibited a similar impact on dexamethasone-mediated antitumor effects, indicating that the inhibitory effects of MSCs against dexamethasone's antitumor effects were associated with LINC00461.

Dexamethasone is one of the most frequently used medications in the treatment of multiple myeloma by preventing the recruitment of blood cells into the lesions where myeloma cells cause damage [9]. The International Myeloma Foundation reported that dexamethasone is one of the most effective medications in the treatment of multiple myeloma [10]. Moreover, the combination of dexamethasone with other medications is also widely used in multiple myeloma [11, 26]. In this study, dexamethasone (10 *μ*M) exhibited antitumor effects against multiple myeloma cell lines, which was supported by (1) dexamethasone (10 *μ*M) treatment decreased the cell viability, (2) dexamethasone (10 *μ*M) treatment increased the cell apoptosis, and (3) dexamethasone (10 *μ*M) treatment led to cells arresting at the G0/G1 stage. These results supported that dexamethasone is an effective medication in the treatment of multiple myeloma.

Numerous studies reported that MSCs play important roles in the occurrence and development of multiple myeloma [15, 17]. When multiple myeloma occurs, MSCs exhibited different functional modes as normal MSCs [19, 27]. The recruitment of MSCs into the tumor sites promotes tumor growth and metastasis in part by regulating the secretion of exosomes and the release of cytokines [19, 27]. In our previous studies, we revealed that MSC-derived exosome promotes the cell proliferation of multiple myeloma, indicating the MSCs might promote the multiple myeloma progression [22]. In recent years, the utilization of MSC-based strategies in the treatment of multiple myeloma has drawn much attention [17, 28–30]. Shimasaki and colleagues found that the survival rate of MSCs is maintained after treatment with dexamethasone [31], indicating the resistance of MSCs to dexamethasone. However, it is still unclear whether MSCs had an impact on dexamethasone-mediated multiple myeloma therapy. In this study, our first aim was to investigate whether the presence of MSCs affected dexamethasone's antitumor effects against multiple myeloma.

Interestingly, our results demonstrated that the presence of MSCs affected the effects of dexamethasone on cell apoptosis, proliferation, and cell cycle distribution. The presence of MSCs increased the cell viability, cell number, and colony number of OPM-2 and RPMI8226 cells as compared to the dexamethasone-treated cells. In addition, MSCs significantly decreased the percentage of apoptotic OPM-2 and RPMI8226 cells by increasing the expressions of BCL-2. The presence of MSCs increased the percentage of cells in the S phase, while it decreased the percentage of cells in the G0/G1 phase. Our results are consistent with the previous findings. For instance, Zhu and colleagues reported that coculturing with MSCs induces the cell proliferation of leukemic cells [32]. Another study found that the presence of MSCs leads to a tumoricidal effect by increasing cell viability in lymphoma cell lines [33]. In our previous studies, the presence of MSCs enhances the cell proliferation of multiple myeloma [22]. In this study, not only MSCs promote multiple myeloma cell growth but also their presence inhibited antitumor effects of dexamethasone against multiple myeloma.

LINC00461 has been reported to have important roles in the occurrence and development of several types of cancers including hepatoma and multiple myeloma [34–36]. Yang and colleagues reported that LINC00461 is upregulated in human gliomas and knockdown of LINC00461 suppresses cell proliferation, migration, and invasion as well as regulates cell cycle distribution by the regulation of MAPK/ERK and PI3K/Akt signaling pathways [35]. Ji and colleagues found that LINC00461 silencing inhibits cell invasion, migration, and proliferation in hepatoma cancer cells and suppresses tumor growth in vivo by the regulation of leucine-rich repeats and immunoglobulin-like domains 2 and miR-149-5p [36]. Our previous findings showed that LINC00461 is highly expressed in multiple myeloma and acts as a sponge for miR-15a/16, thereby promoting cell proliferation of multiple myeloma [22]. More importantly, we also found that LINC00461 is highly expressed in MSC-derived exosomes, indicating that LINC00461 is closely linked with MSCs. Therefore, we further explored if LINC00461 had effects on dexamethasone-mediated multiple myeloma therapy.

Interestingly, the overexpression of LINC00461 exhibited a similar mode as dexamethasone's antitumor effects on multiple myeloma cells. First, LINC00461 overexpression suppressed the inhibitory effects of dexamethasone on cell proliferation. Second, LINC00461 overexpression decreased the percentage of apoptotic cells as compared to the dexamethasone-treated cells. Moreover, LINC00461 overexpression decreased the percentage of cells in the G0/G1 phase and increased the percentage of cells in the S phase. Taken together, we inferred that the inhibitory effects of MSCs on dexamethasone's antitumor effects against multiple myeloma were associated with the overexpression of LINC00461.

## 5. Conclusion

The presence of MSCs affected the effects of dexamethasone on cell proliferation, cell apoptosis, and cell cycle distribution. Interestingly, the overexpression of LINC00461 exhibited a similar mode as dexamethasone's antitumor effects on multiple myeloma cells. Taken together, MSCs inhibited the effects of dexamethasone on multiple myeloma and its inhibitory effects were associated with LINC00461.

## Data Availability

Data could be obtained upon request to the corresponding author.
